# Impact of oral health conditions on the quality of life of preschool children and their families: a cross-sectional study

**DOI:** 10.1186/1477-7525-12-55

**Published:** 2014-04-18

**Authors:** Monalisa Cesarino Gomes, Tassia Cristina de Almeida Pinto-Sarmento, Edja Maria Melo de Brito Costa, Carolina Castro Martins, Ana Flávia Granville-Garcia, Saul Martins Paiva

**Affiliations:** 1 Postgraduate Program in Dentistry, State University of Paraiba (UEPB), 1325/410 Capitão João Alves de Lira, 58428-800 Campina Grande, PB, Brazil; 2 Department of Paediatric Dentistry and Orthodontic, Federal University of Minas Gerais (UFMG), Belo Horizonte, MG, Brazil

**Keywords:** Quality of life, Dental caries, Malocclusion, Tooth injuries, Preschool child

## Abstract

**Background:**

Dental caries, traumatic dental injury (TDI) and malocclusion are common oral health conditions among preschool children and can have both physical and psychosocial consequences. Thus, it is important to measure the impact these on the oral health-related quality of life (OHRQoL) of children. The aim of the present study was to assess the impact of oral health conditions on the OHRQoL of preschool children and their families.

**Methods:**

A preschool-based, cross-sectional study was carried out with 843 preschool children in the city of Campina Grande, Brazil. Parents/caregivers answered the Brazilian Early Childhood Oral Health Impact Scale and a questionnaire addressing socio-demographic data as well as the parent’s/caregiver’s perceptions regarding their child’s health. Clinical exams were performed by three researchers who had undergone a calibration process for the diagnosis of dental caries, TDI and malocclusion (K = 0.83-0.85). Hierarchical Poisson regression was employed to determine the strength of associations between oral health conditions and OHRQoL (α = 5%). The multivariate model was run on three levels obeying a hierarchical approach from distal to proximal determinants: 1) socio-demographic data; 2) perceptions of health; and 3) oral health conditions.

**Results:**

The prevalence of impact from oral health conditions on OHRQoL was 32.1% among the children and 26.2% among the families. The following variables were significantly associated with a impact on OHRQoL among the children: birth order of child (PR = 1.430; 95% CI: 1.045-1.958), parent’s/caregiver’s perception of child’s oral health as poor (PR = 1.732; 95% CI: 1.399-2.145), cavitated lesions (PR = 2.596; 95% CI: 1.982-3.400) and TDI (PR = 1.413; 95% CI: 1.161-1.718). The following variables were significantly associated with a impact on OHRQoL among the families: parent’s/caregiver’s perception of child’s oral health as poor (PR = 2.116; 95% CI: 1.624-2.757), cavitated lesions (PR = 2.809; 95% CI: 2.009-3.926) and type of TDI (PR = 2.448; 95% CI: 1.288-4.653).

**Conclusion:**

Cavitated lesions and TDI exerted a impact on OHRQoL of the preschool children and their families. Parents’/caregivers’ perception of their child’s oral health as poor and the birth order of the child were predictors of a greater impact on OHRQoL.

## Background

Oral health conditions can have a negative impact on the functional, social and psychological wellbeing of young children and their families, causing pain and discomfort for the child [1,2]. Assessing the impact of oral health on the life quality of children can improve communication between patients, parents and the dental team and can provide an outcome measure for clinicians to assess the quality of care. Moreover, the evaluation of oral health-related quality of life (OHRQoL) can assist in the assessment of treatment needs, the prioritisation of care and the evaluation of the outcomes of treatment strategies and initiatives [3-5].

The interest in the assessment of OHRQoL among children has grown in recent years, which is a major improvement, as children in many communities around the world are affected by dental caries, traumatic dental injury (TDI) and malocclusion [2,4-9]. Recent studies carried out in Brazil have shown high prevalence rates of adverse oral conditions, especially in areas with social inequalities, such as the area in which the present study was conducted [10-12].

The Early Childhood Oral Health Impact Scale (ECOHIS) was developed to assess the impact of oral health conditions on the quality of life of preschool children (aged 2 to 5 years) and their families and has been validated in Portuguese for use on Brazilian populations [1,13,14]. This scale is a proxy measure that considers parents/caregivers to be fundamental in the treatment decision-making process and perceptions regarding children’s oral health conditions [1,13]. Moreover evidence in the fields of child development and psychology indicates that children less than six years of age are incapable of accurately recalling day-to-day events after more than 24 hours [15].

Studies evaluating the impact of oral health conditions on OHRQoL are often based on non-randomised [5-7] convenience samples [9,16,17]. Moreover, most studies use dichotomised variables (presence/absence of oral health conditions) [2,9,16,17]. Few studies have stratified the variables based on the severity of TDI and types of malocclusion [5,7] and all studies have evaluated dental caries using the WHO criteria, which does not discriminate cavitated dental caries and the initial stages of dental caries (e.g., white spots). No study in the literature has evaluated the severity of dental caries based on new diagnostic index for dental caries: the International Caries Detection and Assessment System (ICDAS), which is an internationally accepted caries detection system that allows the assessment of initial carious lesions (white spots) on the enamel and active lesions in the dentine. This system is based on the combined knowledge of the clinical appearance of the lesion, whether the lesion is in a plaque stagnation area and tactile sensation (texture) when a round-tipped probe is gently drawn across the surface of the tooth [18,19]. No study has evaluated the impact of white spots and cavitations on OHRQoL. Furthermore, the present investigation is a unique representative, randomised study with a two-stage sampling method and hierarchical analysis to evaluate the impact of the severity of TDI, type of malocclusion, white spots and cavitated dental caries on OHRQoL.

The aim of the present study was to evaluate the impact of oral health conditions on the OHRQoL of preschool children aged three to five years and their families in a representative, preschool-based sample.

## Methods

### Sample characteristics

A cross-sectional study was carried out involving a randomly selected sample of 843 male and female children aged three to five years enrolled in private and public preschools in the city of Campina Grande, Brazil. The participants were selected from a total population of 12,705 children in this age group. Campina Grande (estimated population: 386,000) is an industrialised city in northeast Brazil and is divided into six administrative districts. The city has a Human Development Index of 0.72 [20].

The percentage distribution of three-to-five-year-old preschool children in each administrative district was calculated from information provided by the municipal Board of Education. To ensure representativeness, the sample was stratified according to administrative district and type of institution (two-phase sampling method). Preschools were randomly selected from each administrative district in the first phase and preschool children were randomly selected from each preschool in the second phase. Sample distribution was proportional to the total population enrolled in private and public preschools in each administrative district of the city*.* The sample size was calculated based on a 4% margin of error, a 95% confidence level and a correction factor of 1.2 to compensate for the design effect [21]. As the prevalence of impact on OHRQoL was unknown, a prevalence rate of 50% was considered to increase the power and because this value gives the largest sample regardless of the actual prevalence [22]. Eighteen of the 127 public preschools and 15 of the 122 private preschools were randomly selected. The minimum sample size was estimated at 720 preschool children, to which an additional 20% was added to compensate for possible losses, giving a total sample of 864 preschool children.

The present study received approval from the Human Research Ethics Committee of the State University of Paraíba (Brazil) under process number 00460133000–11 in compliance with Resolution 196/96 of the Brazilian National Health Council. All participants’ rights were protected. Parents/caregivers read and signed a statement of informed consent prior to the children’s participation.

### Eligibility criteria

To be included in the study, the children needed to be between three and five years of age, enrolled in a preschool and free of systemic diseases (based on the reports of the parents/caregivers). Only reports of parents/caregivers were considered for systemic disease; no systemic examination was conducted. The exclusion criteria were the presence of one or more erupted permanent teeth, a history of orthodontic treatment and caregivers not fluent in Brazilian.

### Training and calibration exercise

The training and calibration exercise consisted of two steps (theoretical and clinical). The theoretical step involved a discussion of the criteria for the diagnosis of dental caries, TDI, malocclusion and an analysis of photographs. A specialist in paediatric dentistry (gold standard in this theoretical framework) coordinated this step, instructing three general dentists on how to perform the examination. The clinical step was performed at a randomly selected preschool that was not part of the main sample. Each dentist examined 50 previously selected preschool children between three and five years of age. Inter-examiner agreement was tested by comparing each examiner (K = 0.83 to 0.88). After a seven-day interval, the examinations were performed a second time for the determination of intra-examiner agreement (K = 0.85 to 0.90). Data analysis involved Cohen’s Kappa coefficient on a tooth-by-tooth basis. As the Kappa coefficients were very good [23], the examiners were considered capable of conducting the epidemiological study.

### Pilot study

A pilot study was conducted to test the methodology and comprehension of the questionnaires. The children in the pilot study (n = 40) were not included in the main sample. As there were no misunderstandings regarding the questionnaires or the methodology, no changes to the data collection process were deemed necessary.

### Non-clinical data collection

The acquisition of the non-clinical data involved the administration of the Brazilian version of the Early Childhood Oral Health Impact Scale (B-ECOHIS) and questionnaires addressing socio-demographic data and parents’/caregivers’ perceptions regarding their child’s health. Parents/caregivers were previously contacted to attend a meeting at the preschools, at which they were informed regarding the objectives of the study. Parents/caregivers who agreed to participate signed a statement of informed consent and were then instructed to answer the B-ECOHIS and a questionnaire addressing socio-demographic data. For the B-ECOHIS, the parents/caregivers were instructed to consider the child’s entire lifetime experience of oral health conditions and treatment. All questionnaires were filled out by the parents/caregivers and returned at the end of the meeting.

The B-ECOHIS is used for the evaluation of parents’/caregivers’ perceptions regarding the impact of oral health conditions on the OHRQoL of preschool children and their families. This measure has been employed in previous studies [1,13,14] and is divided into two sections (Child Impact and Family Impact), with a total of six subscales and 13 items. The subscales on the Child Impact section are symptoms (1 item), function (4 items), psychology (2 items) and self-image/social interaction (2 items). The subscales on the Family Impact section are parental distress (2 items) and family function (2 items). Each item has six response options: 0 = never; 1 = hardly ever; 2 = sometimes; 3 = often; 4 = very often; and 5 = “I don’t know”. Questionnaires with two or more unanswered items on the Child Impact section or one or more unanswered item on the Family Impact section were considered incomplete and were excluded from the analysis. The scores are totalled for each section (“don’t know” responses are not counted). The total score ranges from 0 to 36 points on the Child Impact section and 0 to 16 points on the Family Impact section, with higher scores indicating greater impact [1,13]. In the present study, negative impact on child and family OHRQoL was recorded when at least one response of “sometimes”, “often” or “very often” was chosen, whereas responses of “never” and “hardly ever” were considered indicative of an absence of negative impact, as recommended by the creators of the original ECOHIS [15].

The following socio-demographic variables were analysed: sex and age of child; parent’s/guardian’s age; mother’s schooling; number of residents in the home; child’s birth order among siblings; type of preschool (public or private); and monthly household income (categorised based on the monthly minimum salary in Brazil, which was equal to US$312.50).

Parent’s/caregiver’ perceptions regarding their child’s general and oral health status were evaluated based on answers to the following question: In general, how would you describe your child’s general health/oral health? The response options were 1) very good, 2) good, 3) fair, 4) poor and 5) very poor. For statistical purposes, these answers were dichotomised as good (codes 1 and 2) and poor (codes 3, 4 and 5) [2].

### Clinical data collection

After the return of the questionnaires and signed statement of informed consent, clinical examinations were performed at the preschools by three dentists who had undergone the training and calibration exercise. Prior to the exam, each child received a kit containing a toothbrush, toothpaste and dental floss to remove bacterial plaque from the teeth and facilitate the diagnosis. The child was then seated in front of the examiner. Light was provided by a portable lamp positioned on the examiner’s head (Petzl Zoom head lamp, Petzl America, Clearfield, UT, USA). The dentists used individual protection equipment, a sterilised mouth mirror (PRISMA®, São Paulo, SP, Brazil), sterilised Williams probe (WHO-621, Trinity®, Campo Mourão, PA, Brazil) and gauze to dry the teeth.

Dental caries was diagnosed using the International Caries Detection and Assessment System (ICDAS II) [18], which is a scoring system ranging from 0 (absence of dental caries) to 6. Due to the epidemiological nature of the present study, code 1 was not used, as drying of the teeth was performed with gauze rather than compressed air. Code 2 was used for white spots and codes ≥ 3 determined different degrees of cavitations. For statistical purposes, dental caries was dichotomised as absent (code 0) or present (code ≥ 2) [18]. Untreated dental caries was also considered in the evaluation of the impact of cavitated lesions in OHRQoL. This variable was categorised as absent/white spot (codes 0 and 2), cavitated anterior teeth (codes ≥ 3), cavitated posterior teeth (codes ≥ 3), cavitated anterior and posterior teeth (codes ≥ 3). It is noteworthy that code 4 represents those lesions where there are underlying shadows indicating that the carious demineralization has progressed into dentin [18], however not all codes 4 are cavitated but in this study were included as cavitated lesions.

TDI was diagnosed as enamel fracture, enamel + dentine fracture, complicated crown fracture, extrusive luxation, lateral luxation, intrusive luxation and avulsion [24]. A visual inspection was also made of tooth colouration. TDI was recorded in the presence of any type of TDI or tooth discolouration. Malocclusion was recorded in the presence of at least one of the following conditions: increased overbite (>2 mm), increased overjet (>2 mm), anterior open bite, anterior crossbite and posterior crossbite [25,26]. Following the exam, a fluoridated varnish was applied to the teeth and children with dental caries or other dental needs were sent for treatment.

### Statistical analysis

**Figure 1:**
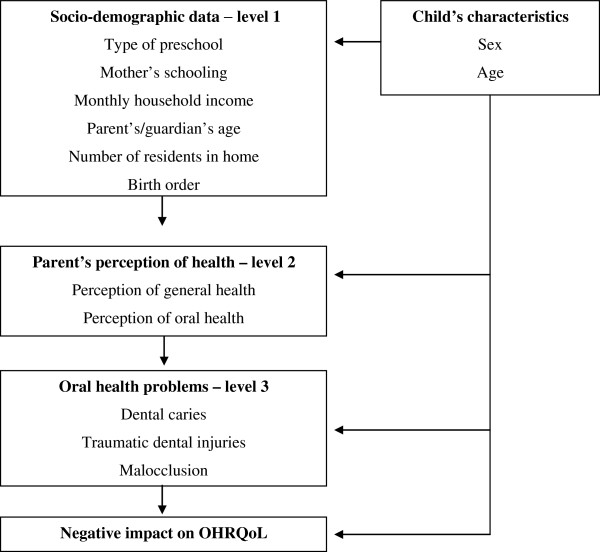
Analysis model used in the study.

Descriptive statistics were first performed to characterise the sample. The chi-square test was used to test associations between oral health conditions and socio-demographic data and the Bonferroni correction was used for variables with more than two categories. Bivariate Poisson regression analysis with robust variance was used to determine associations between the independent variables and negative impact on the OHRQoL of the preschool children and their families (p < 0.05). The multivariate model obeyed a hierarchical approach from distal to proximal determinants: 1) socio-demographic data, 2) parent’s/caregiver’s perception of child’s health and 3) oral health conditions (Figure 1) [27]. The backward stepwise procedure was used to incorporate variables that achieved a p-value < 0.20 in the bivariate analysis as well as variables considered epidemiological determinants on each level. Variables with a p-value < 0.05 in the adjusted analysis were maintained in the final regression model. Interactions among dental caries, TDI and malocclusion were tested using Wald’s test. Variance inflation factors were calculated to determine the existence of collinearity among the predictors in the adjusted model. The data were organised and analysed with the aid of the Statistical Package for Social Sciences (SPSS for Windows, version 20.0, SPSS Inc, Chicago, IL, USA).

## Results

A total of 843 pairs of preschool children and their parents/caregivers participated in the present study, corresponding to 97.5% of the total determined by the sample size calculation. The loss of 21 children was due to a lack of cooperation during the exam (n = 6), incomplete questionnaires (n = 11) and absence from preschool on the days scheduled for the clinical examinations (n = 4).

Table 1 displays the oral health conditions distributed according to the socio-demographic data of the sample. The chi-square test revealed that, among the three oral conditions, only cavitated lesions were significantly associated with age, type of school, mother’s schooling and monthly household income. More children with cavitated lesions were aged five years (56.8%), studied at public preschools (58.6%), had mothers with ≤ eight years of schooling (62.4%) or came from families that earned up to the minimum wage (61.1%) compared to children with white spots or without dental caries. Parents’/caregivers’ perceptions of general and oral health were poor among 81.0% and 66.5% of the sample, respectively.

**Table 1:** Frequency of clinical variables according to socio-demographic data of preschool children analysed

**Variable**	**Dental caries**			**TDI**		**Malocclusion**	
	Absent* n (%)	White spots* n (%)	Cavitations* n (%)	**Absent n (%)**	**Present n (%)**	**Absent n (%)**	**Present n (%)**
**Sex**							
Female	125 (30.7)^A^	82 (20.1)^A^	200 (49.1)^A^	275 (67.9)^A^	130 (32.1)^A^	141 (34.8)^A^	264 (65.2)^A^
Male	158 (36.2)^A^	76 (17.4)^A^	202 (46.3)^A^	278 (64.1)^A^	156 (35.9)^A^	152 (35.0)^A^	282 (65.0)^A^
**Age**							
3 years	107 (38.9)^A^	61 (22.2)^A^	107 (38.9)^B^	176 (64.0)^A^	99 (36.0)^A^	85 (30.9)^A^	190 (69.1)^A^
4 years	103 (30.8)^A^	69 (20.7)^A^	162 (48.5)^B^	226 (68.2)^A^	106 (31.9)^A^	115 (34.6)^A^	217 (65.4)^A^
5 years	73 (31.1)^A^	28 (12.0)^A^	133 (56.8)^B^	151 (65.1)^A^	81 (34.9)^A^	93 (40.1)^A^	139 (59.9)^A^
**Type of preschool**							
Public	111 (24.3)^A^	78 (17.1)^A^	267 (58.6)^B^	305 (67.3)^A^	148 (32.7)^A^	163 (36.0)^A^	290 (64.0)^A^
Private	172 (44.4)^A^	80 (20.7)^A^	135 (34.9)^B^	248 (64.2)^A^	138 (35.8)^A^	130 (33.7)^A^	256 (66.3)^A^
**Mother’s schooling**							
≤ 8 years of study	100 (25.8)^A^	46 (11.9)^A^	242 (62.4)^B^	262 (68.1)^A^	123 (31.9)^A^	133 (34.5)^A^	252 (65.5)^A^
> 8 years of study	183 (40.5)^A^	111 (24.6)^A^	158 (35.0)^B^	289 (64.1)^A^	162 (35.9)^A^	160 (35.5)^A^	291 (64.5)^A^
**Monthly household income**							
≤ 1 minimum salary	108 (24.4)^A^	64 (14.5)^A^	270 (61.1)^B^	299 (68.0)^A^	141 (32.0)^A^	166 (37.7)^A^	274 (62.3)^A^
> 1 minimum salary	157 (43.4)^A^	84 (23.2)^A^	121 (33.4)^B^	229 (63.6)^A^	131 (36.4)^A^	113 (31.4)^A^	247 (68.6)^A^
**TOTAL**	283 (33.6)	158 (18.7)	402 (47.7)	553 (65.9)	286 (34.1)	293 (34.9)	546 (65.1)

*Chi-square test with Bonferroni correction; Different capital letters denote significantly different results (*p* < 0.05).

The prevalence of negative impact on OHRQoL was 32.1% among the children and 26.2% among the families. Scores of 0 (floor effect) were found on 58.6% and 69.6% of the Child Impact and Family Impact sections of the B-ECOHIS, respectively (i.e., 58.6% and 69.6% of the parents/caregivers reported no impacts). No ceiling effect was found for either section (i.e., scores of 36 and 16 on the Child and Family Impact sections, respectively). The maximum score was 31 on the Child Impact section and 14 on the Family Impact section (Table 2). Table 2 also displays the mean, standard deviation, median, minimum and maximum total B-ECOHIS scores and subscales scores. The items with the highest means were “reported pain”, “felt guilty” and “been upset”.

**Table 2:** Prevalence of impact on oral health-related quality of life and B-ECOHIS scores among preschool children

**Subscales, Items**	**SCORE Mean ± SD**	**Median**	**Minimum-Maximum**	**Impact N (%)**
**ECOHIS total (0–52)**	3.60 ± 6.10	0	0 - 38	349 (41.4)
**Child Impact**	2.41 ± 4.41	0	0 - 31	271 (32.1)
Reported pain	0.61 ± 0.99	0	0 - 4	195 (23.1)
Had difficulty drinking hot or cold beverages	0.36 ± 0.82	0	0 - 4	110 (13.1)
Had difficulty eating some foods	0.37 ± 0.85	0	0 - 4	112 (13.3)
Had difficulty pronouncing words	0.22 ± 0.72	0	0 - 4	66 (7.8)
Missed preschool, day care or school	0.13 ± 0.49	0	0 - 3	34 (4.1)
Had trouble sleeping	0.20 ± 0.64	0	0 - 4	56 (6.6)
Been irritable or frustrated	0.31 ± 0.78	0	0 - 4	95 (11.3)
Avoided smiling or laughing	0.11 ± 0.49	0	0 - 4	26 (3.1)
Avoided talking	0.10 ± 0.45	0	0 - 4	27 (3.2)
**Family Impact**	1.23 ± 2.31	0	0 - 14	221 (26.2)
Been upset	0.41 ± 0.92	0	0 - 4	126 (15.0)
Felt guilty	0.49 ± 0.97	0	0 - 4	157 (18.7)
Taken time off work	0.17 ± 0.59	0	0 - 4	56 (6.7)
Financial impact	0.16 ± 0.60	0	0 - 4	46 (5.4)

In the final hierarchical Poisson regression model, the following variables were significantly associated with a negative impact on OHRQoL among the children: child’s order of birth, parent’s/caregiver’s perception of child’s oral health as poor, cavitated dental caries and TDI (Table 3). The following variables were significantly associated with a negative impact on OHRQoL among the families: parent’s/caregiver’s perception of child’s oral health as poor, cavitated dental caries and greater severity of TDI (Table 4).

**Table 3:** Hierarchical Poisson regression for impact on OHRQoL of children and independent variables

**Variable**	**Impact on child’s OHRQoL**		**Bivariate**		**Multivariate**	
	**Present n (%)**	**Absent n (%)**	**Unadjusted**		**Adjusted**	
			PR*		**PR †**	
			**p-value**		**p-value**	
			**(95% CI)**		**(95% CI)**	
**Sex**						
Female	132 (32.4)	275 (67.6)	0.864	1.017 (0.836-1.238)	-	-
Male	139 (31.9)	297 (68.1)		1.00	-	-
**Age**						
3 years	71 (25.8)	204 (74.2)		1.00	-	-
4 years	95 (28.4)	239 (71.6)	0.470	1.102 (0.847-1.433)	-	-
5 years	105 (44.9)	129 (55.1)	<0.001	1.738 (1.360-2.222)	-	-
**1**^**st **^**level**						
**Type of preschool**						
Public	171 (37.5)	285 (62.5)	<0.001	1.451 (1.181-1.784)	-	-
Private	100 (25.8)	287 (74.2)		1.00	-	-
**Mother’s schooling**						
≤ 8 years of study	153 (39.4)	235 (60.6)	<0.001	1.510 (1.239-1.841)	-	-
> 8 years of study	118 (26.1)	334 (73.9)		1.00	-	-
**Monthly household income**						
≤ 1 minimum salary	177 (40.0)	265 (60.0)	<0.001	1.686 (1.357-2.094)	-	-
> 1 minimum salary	86 (23.8)	276 (76.2)		1.00	-	-
**Parent’s/guardian’s age**						
≤ 30 years	139 (32.9)	283 (67.1)	0.456	1.079 (0.883-1.319)	-	-
> 30 years	123 (30.5)	280 (69.5)		1.00	-	-
**Number of residents in home**						
< 6	214 (30.6)	485 (69.4)		1.00	-	-
≥ 6	53 (41.1)	76 (58.9)	0.014	1.342 (1.061-1.697)	-	-
**Birth order**						
Only child	60 (22.8)	203 (77.2)		1.00		1.00
Oldest child	50 (40.7)	73 (59.3)	<0.001	1.782 (1.309-2.425)	0.025	1.430 (1.045-1.958)
Middle child	38 (36.5)	66 (63.5)	0.006	1.602 (1.143-2.243)	0.316	1.183 (0.852-1.641)
Youngest child	121 (34.7)	228 (65.3)	0.002	1.520 (1.166-1.981)	0.106	1.228 (0.957-1.575)
**2**^**nd **^**level**						
**Perception of general health**						
Good	201 (29.6)	479 (70.4)		1.00	-	-
Poor	70 (44.0)	89 (56.0)	<0.001	1.489 (1.207-1.838)	-	-
**Perception of oral health**						
Good	120 (21.4)	440 (78.6)		1.00		1.00
Poor	151 (53.5)	131 (46.5)	<0.001	2.499 (2.062-3.029)	<0.001	1.732 (1.399-2.145)
**3**^**rd **^**level**						
**Dental caries**						
Absent	46 (16.3)	237 (83.7)		1.00	-	-
Present	225 (40.2)	335 (59.8)	<0.001	2.472 (1.862-3.281)	-	-
**Cavitated lesions**						
Absent/white spots	77 (17.5)	364 (82.5)		1.00		1.00
Cavitated anterior teeth	17 (29.3)	41 (70.7)	0.024	1.679 (1.072-2.628)	0.146	1.422 (0.885-2.287)
Cavitated posterior teeth	82 (44.6)	102 (55.4)	<0.001	2.552 (1.970-3.307)	<0.001	2.011 (1.518-2.664)
Cavitated anterior and posterior teeth	95 (59.4)	65 (40.6)	<0.001	3.401 (2.675-4.323)	<0.001	2.596 (1.982-3.400)
**TDI**						
Absent	168 (30.4)	385 (69.6)		1.00		1.00
Present	100 (35.0)	186 (65.0)	0.173	1.151 (0.940-1.409)	0.001	1.413 (1.161-1.718)
**Type of TDI**						
Discolouration	38 (39.2)	59 (60.8)	0.071	1.285 (0.979-1.688)	-	-
Avulsion and/or luxation	4 (36.4)	7 (63.6)	0.661	1.193 (0.542-2.628)	-	-
Enamel + dentine fracture	16 (38.1)	26 (61.9)	0.276	1.250 (0.836-1.868)	-	-
Enamel fracture and without trauma	210 (30.5)	479 (69.5)		1.00	-	-
**Malocclusion**						
Absent	95 (32.4)	198 (67.6)	0.827	1.023 (0.833-1.258)	-	-
Present	173 (31.7)	373 (68.3)		1.00	-	-
**Anterior crossbite**						
Absent	256 (31.5)	557 (68.5)		1.00	-	-
Present	9 (39.1)	14 (60.9)	0.413	1.243 (0.739-2.090)	-	-
**Anterior open bite**						
Absent	201 (31.1)	446 (68.9)		1.00	-	-
Present	58 (32.8)	119 (67.2)	0.663	1.055 (0.830-1.341)	-	-
**Posterior crossbite**						
Absent	229 (31.3)	503 (68.7)		1.00	-	-
Unilateral	30 (32.3)	63 (67.7)	0.848	1.031 (0.754-1.411)	-	-
Bilateral	5 (55.6)	4 (44.4)	0.058	1.776 (0.980-3.217)	-	-
**Increased overbite**						
Absent	224 (33.6)	442 (66.4)	0.009	1.518 (1.112-2.073)	-	-
Present	35 (22.2)	123 (77.8)		1.00	-	-
**Increased overjet**						
Absent	144 (30.6)	326 (69.4)		1.00	-	-
Present	121 (33.1)	245 (66.9)	0.455	1.079 (0.884-1.317)	-	-

*Unadjusted Poisson regression for independent variables and impact on child’s quality of life.

**Variables incorporated in multivariate model (p < 0.20): sex, age, type of preschool, mother’s schooling, monthly household income, number of residents in home, child’s birth order, parent’s/caregiver’s perception of child’s general health, parent’s/caregiver’s perception of child’s oral health, dental caries, untreated dental caries, traumatic dental injury, type of traumatic dental injury, malocclusion, posterior crossbite and increased overbite.

***For dental caries, the cut-off point was code ≥ 2; for cavitated lesions, absent/white spots (codes ≤ 2) and cavitated tooth (codes ≥ 3) were considered.

†Hierarchical Poisson regression: Level 1 adjusted for child’s characteristics and socio-demographic variables; Level 2 adjusted for child’s characteristics, socio-demographic variables and parent’s/caregiver’s perception of child’s health; Level 3 adjusted for child’s characteristics, socio-demographic variables, parent’s/caregiver’s perception of child’s health and oral health problems (dental caries, traumatic dental injuries and malocclusion).

**Table 4:** Hierarchical Poisson regression for impact on OHRQoL of family and independent variables

**Variable**	**Impact on family’s OHRQoL**		**Bivariate**		**Multivariate**	
	**Present n (%)**	**Absent n (%)**	**Unadjusted**		**Adjusted**	
			PR*		**PR †**	
			**p-value**		**p-value**	
			**(95% CI)**		**(95% CI)**	
**Sex**						
Female	106 (26.0)	301 (74.0)		1.00	-	-
Male	115 (26.4)	321 (73.6)	0.913	1.013 (0.807-1.270)	-	-
**Age**						
3 years	66 (24.0)	209 (76.0)		1.00	-	-
4 years	85 (25.4)	249 (74.6)	0.681	1.060 (0.802-1.402)	-	-
5 years	70 (29.9)	164 (70.1)	0.133	1.246 (0.935-1.662)	-	-
**1**^**st **^**level**						
**Type of preschool**						
Public	131 (28.7)	325 (71.3)	0.074	1.235 (0.980-1.557)	-	-
Private	90 (23.3)	297 (76.7)		1.00	-	-
**Mother’s schooling**						
≤ 8 years of study	117 (30.2)	271 (69.8)	0.019	1.311 (1.045-1.644)	-	-
> 8 years of study	104 (23.0)	348(77.0)		1.00	-	-
**Monthly household income**						
≤ 1 minimum salary	132 (29.9)	310 (70.1)	0.023	1.318 (1.039-1.673)	-	-
> 1 minimum salary	82 (22.7)	280 (77.3)		1.00	-	-
**Parent’s/guardian’s age**						
≤ 30 years	120 (28.4)	302 (71.6)	0.080	1.232 (0.975-1.557)	-	-
> 30 years	93 (23.1)	310 (76.9)		1.00	-	-
**Number of residents in home**						
< 6	176 (25.2)	523 (74.8)		1.00	-	-
≥ 6	43 (33.3)	86 (66.7)	0.046	1.324 (1.005-1.744)	-	-
**Birth order**						
Only child	55 (20.9)	208 (79.1)		1.00	-	-
Oldest child	39 (31.7)	84 (68.3)	0.020	1.516 (1.068-2.152)	-	-
Middle child	27 (26.0)	77 (74.0)	0.290	1.241 (0.832-1.853)	-	-
Youngest child	98 (28.1)	251 (71.9)	0.046	1.343 (1.006-1.792)	-	-
**2**^**nd **^**level**						
**Perception of general health**						
Good	157 (23.1)	523 (76.9)		1.00	-	-
Poor	61 (38.4)	98 (61.6)	<0.001	1.662 (1.307-2.113)	-	-
**Perception of oral health**						
Good	88 (15.7)	472 (84.3)		1.00		1.00
Poor	133 (47.2)	149 (52.8)	<0.001	3.001 (2.389-3.770)	<0.001	2.116 (1.624-2.757)
**3**^**rd **^**level**						
**Dental caries**						
Absent	35 (12.4)	248 (87.6)		1.00	-	-
Present	186 (33.2)	374 (66.8)	<0.001	2.686 (1.928-3.742)	-	-
**Cavitated lesions**						
Absent/white spots	55 (12.5)	386 (87.5)		1.00		1.00
Cavitated anterior teeth	14 (24.1)	44 (75.9)	0.013	1.935 (1.152-3.252)	0.079	1.586 (0.949-2.651)
Cavitated posterior teeth	73 (39.7)	111 (60.3)	<0.001	3.181 (2.345-4.315)	<0.001	2.380 (1.679-3.372)
Cavitated anterior and posterior teeth	79 (49.4)	81 (50.6)	<0.001	3.959 (2.954-5.306)	<0.001	2.809 (2.009-3.926)
**TDI**						
Absent	143 (25.9)	410 (74.1)		1.00	-	-
Present	74 (25.9)	212 (74.1)	0.996	1.001 (0.786-1.274)	-	-
**Type of TDI**						
Discolouration	32 (33.0)	65 (67.0)	0.080	1.322 (0.968-1.805)	0.054	1.326 (0.995-1.766)
Avulsion and/or luxation	4 (36.4)	7 (63.6)	0.352	1.457 (0.660-3.217)	0.006	2.448 (1.288-4.653)
Enamel + dentine fracture	9 (21.4)	33 (78.6)	0.614	0.858 (0.474-1.554)	0.877	1.048 (0.582-1.885)
Enamel fracture and without trauma	172 (25.0)	517 (75.0)		1.00		1.00
**Malocclusion**						
Absent	81 (27.6)	212 (72.4)	0.386	1.110 (0.877-1.405)	-	-
Present	136 (24.9)	410 (75.1)		1.00	-	-
**Anterior crossbite**						
Absent	207 (25.5)	606 (74.5)		1.00	-	-
Present	9 (39.1)	14 (60.9)	0.107	1.537 (0.911-2.593)	-	-
**Anterior open bite**						
Absent	166 (25.7)	481 (74.3)	0.950	1.009 (0.759-1.341)	-	-
Present	45 (25.4)	132 (74.6)		1.00	-	-
**Posterior crossbite**						
Absent	548 (74.9)	184 (25.1)		1.00	-	-
Unilateral	27 (29.0)	66 (71.0)	0.408	1.155 (0.821-1.625)	-	-
Bilateral	5 (55.6)	4 (44.4)	0.009	2.210 (1.216-4.017)	-	-
**Increased overbite**						
Absent	185 (27.8)	481 (72.2)	0.006	1.688 (1.164-2.449)	-	-
Present	26(16.5)	132(83.5)		1.00	-	-
**Increased overjet**						
Absent	128 (27.2)	342 (72.8)	0.298	1.133 (0.896-1.432)	-	-
Present	88 (24.0)	278 (76.0)		1.00	-	-

*Unadjusted Poisson regression for independent variables and impact on family’s quality of life.

**Variables incorporated in multivariate model (p < 0.20): sex, age, type of preschool, mother’s schooling, monthly household income, parent’s/guardian’s age, number of residents in home, child’s birth order, parent’s/caregiver’s perception of child’s general health, parent’s/caregiver’s perception of child’s oral health, dental caries, untreated dental caries, traumatic dental injury, type of traumatic dental injury, malocclusion, anterior crossbite, posterior crossbite and increased overbite.

***For dental caries, the cut-off point was code ≥ 2; for cavitated lesions, absent/white spots (codes ≤ 2) and cavitated tooth (codes ≥ 3) were considered.

†Hierarchical Poisson regression: Level 1 adjusted for child’s characteristics and socio-demographic variables; Level 2 adjusted for child’s characteristics, socio-demographic variables and parent’s/caregiver’s perception of child’s health; Level 3 adjusted for child’s characteristics, socio-demographic variables, parent’s/caregiver’s perception of child’s health and oral health problems (dental caries, traumatic dental injuries and malocclusion).

## Discussion

The present study evaluated the impact of dental caries, TDI and malocclusion on the OHRQoL of preschool children using the Brazilian Portuguese version of the ECOHIS. The occurrence of cavitated lesions and TDI was found to cause a negative impact on the OHRQoL of preschool children and their families, whereas different types of malocclusion did not have this effect. Moreover, this is the first study to perform a hierarchical approach, which stratifies the impact of the severity of TDI, different types of malocclusion, different stages of dental caries and the teeth affected as risk factors that exert an influence on quality of life. Such an approach allows an analysis of interrelationships among factors rather than making the strict statistical associations commonly found in multivariate methods [27].

Cavitated lesions were associated with OHRQoL among the children and families due to the fact that parents/caregivers recognise an oral health problem when it becomes evident or when it is manifested in the form of pain [28,29]. Indeed, the parents/caregivers reported greater impact on the items “reported pain”, “difficulty drinking hot or cold beverages”, “difficulty eating some foods” and “been irritable”. These findings indicate symptoms related to more serious oral health problems, such as untreated dental caries [30]. Severe dental caries can result in parents/caregivers missing days of work and greater financial expenditures as well as feelings of guilt, with a consequent negative impact on the OHRQoL of the family [17,28]. Moreover, the greater participation of women in the job market and consequent reduction in the role mothers play in raising their children [31] may have contributed to the greater frequency of the items “felt guilty” and “been upset”, as suggested in previous studies [2,8,17]. It should be stressed that dental caries was diagnosed in the present study using the criteria of the ICDAS-II, which detects the early manifestations of this condition [18]. This may explain why the presence of dental caries when considering data on white spots was not associated with OHRQoL, as such lesions often go unperceived by parents/caregivers and do not cause pain. Cavitated teeth have been associated to a negative impact on OHRQoL in previous investigations [2,5,6,17,29], although the location of the lesions was not evaluated in the studies cited. Cavitated lesions on anterior teeth were not associated with a negative impact on OHRQoL, likely due to the nature of these lesions, which were not very severe, and the lesser importance given to aesthetics in the age group analysed [32]. However, when posterior teeth and both posterior and anterior teeth were analysed, significant associations were found with a negative impact on OHRQoL, possibly because cavities on posterior teeth are generally more severe and associated with reports of pain and difficulty eating [28,30].

Cavitated lesions were associated with socio-demographic variables (lower income, lower educational level of the mother, enrolment at a public preschool and children aged five years) in comparison to children with white spots or without dental caries. This demonstrates the importance of socio-demographic data regarding the use of dental services [33,34]. Some studies have found a greater frequency of impact on the OHRQoL of preschool children from families with a lower socioeconomic status [2,6,17]. However, no socioeconomic variable remained associated with the negative impact on quality of life in the present investigation, which is in agreement with data reported in a previous study [5]. This finding suggests that oral health conditions can exert an impact on the quality of life of children regardless of one’s socioeconomic status.

While a previous investigation found an association between malocclusion and impact on OHRQoL [5], the majority of studies found no such association [2,7,9,17,35], which is in agreement with the present findings. However, TDI had a negative impact on the OHRQoL of the preschool children and cases of avulsion and/or luxation were predictors of a negative impact on the OHRQoL of the families. This type of oral health problem may require a considerable amount of time on the part of the family due to the urgency of relieving the pain symptoms and the limitations that may arise [5,9,28]. Parents/caregivers generally perceive a negative impact on quality of life when clinical signs are involved, such as tooth discolouration, which can exert a psychosocial impact on the child [8]. However, some studies have found no association between TDI and OHRQoL [2,8,17]. This may be due to the type of TDI considered in the analysis, as negative impact generally only occurs in more serious cases [7,9,28].

Birth order of the child was a predictor of a greater negative impact on OHRQoL among the children, likely because financial resources and attention from parents/caregivers are shared among the siblings as more children are born into the family [36,37]. Indeed, a greater frequency of dental caries is found among children in large families [38].

Parents’/caregivers’ perception of their child’s oral health was associated with a negative impact on the OHRQoL of both the children and families. Perceptions of parents/caregivers regarding their child’s oral health plays an important role in the determination of the negative impact on OHRQoL [39], as the health of preschool children depends on parental/caregiver knowledge regarding health care [40].

The present study has the limitations inherent to the cross-sectional design and the answers on the questionnaires may have been subject to information bias. However, a number of measures were taken to diminish the occurrence of such bias, such as the use of a validated questionnaire and the execution of a pilot study. Thus, it is possible to extrapolate the findings, since the present investigation was a representative, preschool-based study. Longitudinal studies are needed to clarify the relationship of causality and allow establishment of public policies aimed at reducing the impact of oral health conditions on the OHRQoL of preschool children and their families.

## Conclusion

Cavitated lesions on anterior and posterior teeth, traumatic dental injuries and parents’/caregivers’ perception of their child’s oral health as poor are determinants of a negative impact on the OHRQoL of preschool children and their families. While white spots were not associated with impact on OHRQoL, it is important to treat such cases to prevent the progression to cavitation.

## Abbreviations

OHRQoL: Oral health-related quality of life; TDI: Traumatic dental injury; ECOHIS: Early Childhood Oral Health Impact Scale; B-ECOHIS: Brazilian version of the Early Childhood Oral Health Impact Scale; ICDAS: International Caries Detection and Assessment System.

## Competing interests

The authors declare that they have no competing interests.

## Authors' contributions

MCG was responsible for the analysis and interpretation of the data, helped the statistical analysis and drafted the manuscript. TCAPS was responsible for the conception and study design, acquisition and interpretation of data. EMMBC performed data acquisition and drafted the manuscript. CCM performed the analysis and interpretation of the data and a critical review of the manuscript. AFGG was responsible for conception design, analysis and interpretation of the data and a critical review of the manuscript. SMP was responsible for the conception and study design and performed the final critical review. All authors read and approved the final manuscript.
